# An optogenetic method for investigating presynaptic molecular regulation

**DOI:** 10.1038/s41598-021-90244-0

**Published:** 2021-05-31

**Authors:** Yuni Kay, Bruce E. Herring

**Affiliations:** 1 Neuroscience Graduate Program, University of Southern California, Los Angeles, CA 90089 USA; 2 Department of Biological Sciences, Neurobiology Section, Dornsife College of Letters, Arts and Sciences, University of Southern California, Los Angeles, CA 90089 USA

**Keywords:** Neuroscience, Cellular neuroscience, Molecular neuroscience

## Abstract

While efficient methods are well established for studying postsynaptic protein regulation of glutamatergic synapses in the mammalian central nervous system, similarly efficient methods are lacking for studying proteins regulating presynaptic function. In the present study, we introduce an optical/electrophysiological method for investigating presynaptic molecular regulation. Here, using an optogenetic approach, we selectively stimulate genetically modified presynaptic CA3 pyramidal neurons in the hippocampus and measure optically-induced excitatory postsynaptic currents produced in unmodified postsynaptic CA1 pyramidal neurons. While such use of optogenetics is not novel, previous implementation methods do not allow basic quantification of the changes in synaptic strength produced by genetic manipulations. We find that incorporating simultaneous recordings of fiber volley amplitude provides a control for optical stimulation intensity and, as a result, creates a metric of synaptic efficacy that can be compared across experimental conditions. In the present study, we utilize our new method to demonstrate that inhibition of synaptotagmin 1 expression in CA3 pyramidal neurons leads to a significant reduction in Schaffer collateral synapse function, an effect that is masked with conventional electrical stimulation. Our hope is that this method will expedite our understanding of molecular regulatory pathways that govern presynaptic function.

## Introduction

Highly specific, efficient, and quantitative methods currently exist for studying the influence of postsynaptic proteins on glutamatergic synapse function in the mammalian central nervous system. For example, viral and biolistic approaches may be used to restrict genetic manipulations to postsynaptic neurons, and the consequences of such manipulations may be measured by electrophysiological recordings from individual transduced/transfected postsynaptic neurons during electrical stimulation of unmodified presynaptic neurons^1–5^. However, comparable methods are sorely lacking for the study of proteins regulating presynaptic glutamatergic synapse function. While viral methods may be used to restrict genetic manipulations to presynaptic neurons, the consequences of such manipulations on synaptic function require electrophysiological recordings from postsynaptic neurons. Even with high titer viruses, a mixed population of transduced/untransduced presynaptic neurons are present. As a result, electrical stimulation of presynaptic axons in this preparation produces neurotransmitter release from both genetically modified and unmodified neurons which precludes an accurate measurement of the impact the presynaptic genetic modification has on synaptic efficacy in electrophysiological recordings from postsynaptic neurons.

At present, the only way to ensure genetic modification of all presynaptic neurons is to engineer knockout/knockin mouse lines. However, traditional germline approaches result in the genetic modification of both pre- and postsynaptic neurons often making it difficult to separate potential pre- and postsynaptic roles for the proteins being studied. To limit genetic modification to presynaptic neurons, conditional knockout/knockin mice must be engineered and then crossed to specific driver lines. This approach can be extremely costly and time-consuming, especially when paralogous proteins need to be considered.

Difficulty in studying the regulation of presynaptic function has motivated the recent development of new methods to identify changes in presynaptic function. For example, optical tools have now been developed that fluoresce upon binding to glutamate and may be expressed in either pre- or postsynaptic neurons^6^. While certainly useful in potentially detecting alterations in glutamate release, it can be difficult to determine the physiological relevance of such changes (e.g. whether such changes ultimately produce measurable alterations in the number of glutamate receptors that are activated postsynaptically). Recognizing our current lack of an efficient and rigorous approach to study presynaptic protein function at native mammalian glutamatergic synapses in the CNS, we describe in the present study an optical/electrophysiological method for investigating presynaptic molecular regulation.

In this study, we use an optogenetic approach to selectively stimulate presynaptic CA3 pyramidal neurons expressing an RNAi against our protein of interest and measure optically-induced excitatory postsynaptic currents (oEPSCs) produced in unmodified postsynaptic CA1 pyramidal neurons using a conventional whole-cell patch clamping technique. While such usage of optogenetics-driven genetic manipulation in presynaptic studies is not novel^7,8^, previous implementation methods do not allow basic quantification of the changes in synaptic strength produced by the genetic manipulations. This is because in order to make meaningful comparisons of synaptic strengths across experimental conditions, a quantitative measurement of stimulus strength is required to ensure a comparable number of CA3 pyramidal neurons is stimulated in each condition. With our novel method, we resolve this problem by simultaneously measuring fiber volleys from CA3 pyramidal neuron axons (Schaffer collaterals) while recording from CA1 pyramidal neurons. Fiber volley amplitude is determined by the number of CA3 pyramidal neurons firing action potentials. Thus, we are able to derive a metric of synaptic efficacy for each recording that can be compared across conditions by obtaining a CA1-oEPSC/CA3-fiber volley (FV) amplitude ratio.

To assess the accuracy of this approach, we determined whether changes in oEPSC/FV amplitude ratios match increases in glutamatergic synapse density. By comparing hippocampal slices cultured from two different developmental timepoints during a period of robust synaptogenesis, we observe a significant increase in dendritic spine density with increased animal age. We find that our method accurately matches this change by comparing NMDAR-oEPSC/FV amplitudes ratios between the two age groups. Additionally, the present study utilizes the new method to study an important and readily studied presynaptic protein that has not previously been studied at the CA3-CA1 synapse: synaptotagmin 1. Using our method, we find that there is a significant reduction in CA1 current amplitude following Syt1 knockdown in CA3 pyramidal neurons. This phenotype is masked when Schaffer collaterals are stimulated electrically. The significant reduction is only discernible using optical stimulation, demonstrating the critical need for incorporating optogenetics in our approach. Thus, our method of presynaptic interrogation represents a simple, cost-effective, and time-efficient approach that does not require generating new transgenic mouse lines for every protein of interest.

## Results

### Method Setup

**Figure 1:**
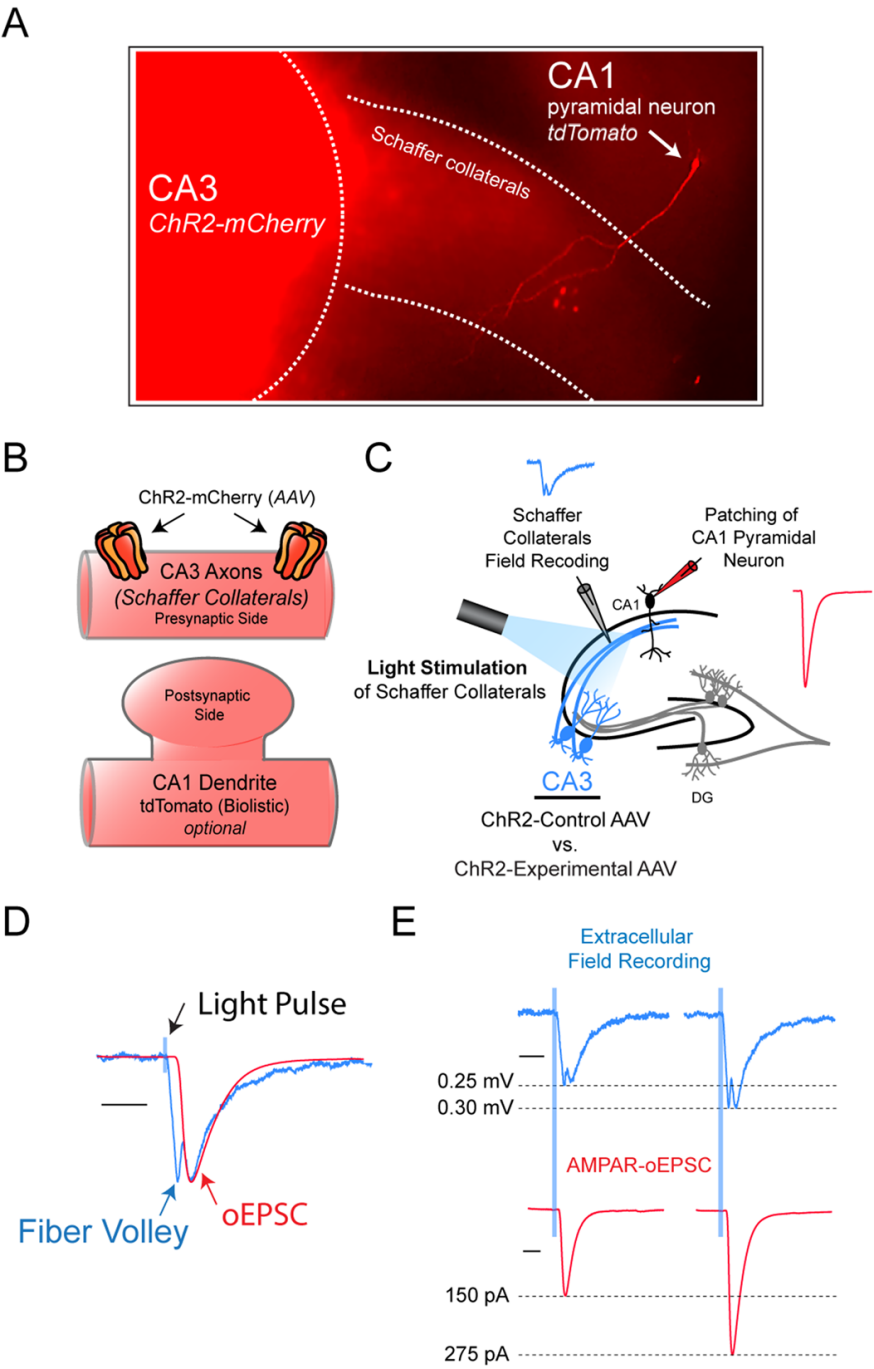
Method setup. (**A**) Image of rat hippocampal slice culture setup for recording CA1 pyramidal neuron currents with optical stimulation of Schaffer collaterals. The CA3 region was injected with an AAV expressing ChR2 tagged with mCherry, and mCherry fluorescence can be seen in both the CA3 pyramidal neuron cell bodies and axons (Schaffer collaterals). A biolistically transfected CA1 pyramidal neuron is also visible by its tdTomato fluorescence. (**B**) Schematic illustration of a CA3-CA1 synapse in our setup. ChR2-mCherry is expressed in the presynaptic Schaffer collaterals via AAV virus, and biolistically-transfected tdTomato is optionally expressed postsynaptically in the CA1 pyramidal neuron. (**C**) Schematic illustration of experimental setup. Virally transduced CA3 pyramidal neurons expressing ChR2 are stimulated optically using 470 nm blue light to evoke a postsynaptic response in CA1 pyramidal neurons. Whole-cell recordings of a CA1 pyramidal neuron were made simultaneously with extracellular field recordings in the Schaffer Collaterals. (**D**) Optically-induced extracellular field recording trace (blue) merged with postsynaptic whole-cell recording trace (red), verifying that the two traces align in time and illustrating the presynaptic fiber volley. Scale bar: 20 ms. (**E**) Presynaptic fiber volley amplitude and postsynaptic current amplitude scale with light stimulus intensity. Scale bar: 20 ms.

Our method of presynaptic molecular interrogation combines optogenetics, postsynaptic whole-cell patch clamping, and extracellular field recordings. The setup begins with the expression of channelrhodopsin (ChR2) in a population of presynaptic neurons. Here, to study the CA3-CA1 synapse, we injected an AAV expressing ChR2 tagged with mCherry directly into the CA3 region of cultured rat hippocampal slices. We verified successful viral transduction via mCherry epifluorescence in the CA3 region and particularly in the Schaffer collaterals (Fig. 1A). We also biolistically transfected a CA1 pyramidal neuron with tdTomato in order to visually demonstrate the hippocampal slice setup where the Schaffer collaterals expressing ChR2-mCherry overlap with dendrites of CA1 pyramidal neurons (Fig. 1A,B). Schaffer collaterals expressing ChR2 were optically stimulated to evoke an AMPA receptor (AMPAR)-mediated oEPSC in CA1 pyramidal neurons. Whole-cell recordings of a CA1 pyramidal neuron were made simultaneously with extracellular field recordings from Schaffer collaterals (Fig. 1C). By aligning the optically-induced extracellular field recording trace with the AMPAR-oEPSC, we verified that the postsynaptic responses (field EPSP and oEPSC) were aligned in time, and that we could isolate the presynaptic fiber volley (Fig. 1D). Furthermore, we found that with increased optical stimulation strength, there was a corresponding increase in both presynaptic fiber volley amplitude and AMPAR-oEPSC amplitude, demonstrating that these measurements scale with light intensity (Fig. 1E). Fiber volley amplitude represents the number of CA3 pyramidal neurons firing action potentials and, as a result, can be used as a readout of Schaffer collateral stimulation strength. To create a metric of synaptic efficacy, CA1-oEPSC amplitude was then divided by fiber volley amplitude to generate a normalized CA1-oEPSC/CA3-FV amplitude ratio that provides the amount of postsynaptic current produced in a neuron by a given amount of presynaptic stimulation.

### Method Validation 1: Increased NMDAR-oEPSC/FV amplitude ratio mirrors glutamatergic synaptogenesis

**Figure 2:**
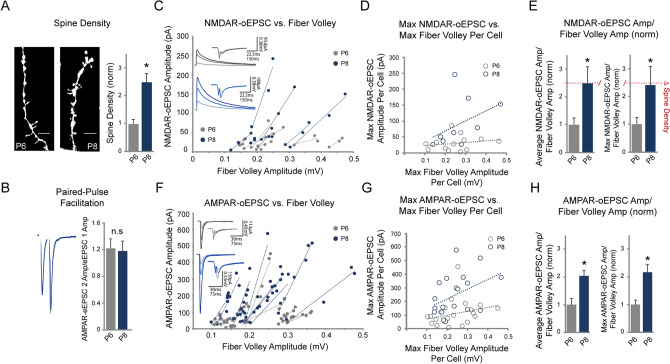
Increased NMDAR-oEPSC/FV amplitude ratio mirrors glutamatergic synaptogenesis**. (A**) Hippocampal slice cultures from P8 rats have increased spine density compared to those from P6 rats. (Left) Representative CA1 dendritic spine images in P6 vs. P8 slices, neurons transfected with GFP. (Right) Bar graph showing averaged spine density in P6 vs. P8 slices normalized to P6 slices (P6: 0.12 ± 0.019 spines/µM, n = 11, P8: 0.29 ± 0.039 spines/µM, n = 14, *p* = 0.001, Student’s t test). For (**B**)–(**H**), Wilcoxon rank sum test was used; * p < 0.05. (**B**) Representative traces (left) and mean ± SEM paired-pulse facilitation ratios for P6 and P8 CA1 pyramidal neurons (right) (P6: n = 11; P8: n = 7, *p* = 1, n.s., not significant). (**C**) NMDAR-oEPSC’s plotted as a function of fiber volley amplitude over a range of stimulation strengths (range = 3 + light intensity levels per cell); measurements from each cell were fitted with a linear regression line. Inset representative traces demonstrate increasing fiber volley amplitude and increasing NMDAR-oEPSC amplitude. (**D**) Max NMDAR-oEPSC/FV amplitude measurements, taken at maximum optical stimulation, for each cell in P6 and P8 slices. Each point represents the Max NMDAR-oEPSC/FV amplitude of one cell. Measurements from each group (P6 vs. P8) were fitted with a linear regression line. (**E**) (Left) Averaged and normalized NMDAR-oEPSC/FV amplitude ratio in P6 vs. P8 slices (P6: n = 7, P8: n = 8, *p* = 0.02). (Right) Averaged and normalized Max NMDAR-oEPSC/FV ratio in P6 vs. P8 slices (P6: n = 10, P8: n = 13, *p* = 0.035). The red dotted line shows fold change in dendritic spine density in (A). (**F**) AMPAR-oEPSC’s plotted as a function of fiber volley amplitude over a range of stimulation strengths (range = 3 + light intensity levels per cell). Measurements from each cell were fitted with a linear regression line. Inset representative traces demonstrate increasing fiber volley amplitude and increasing AMPAR-oEPSC amplitude. (**G**) Max AMPAR-oEPSC/FV amplitude measurements, taken at maximum optical stimulation, for each cell in P6 and P8 slices. Each point represents the Max AMPAR-oEPSC/FV amplitude ratio of one cell. Measurements from each group (P6 vs. P8) were fitted with a linear regression line. (**H**) (Left) Averaged and normalized AMPAR-oEPSC/FV amplitude ratio in P6 vs. P8 slices (P6: n = 10, P8: n = 11, *p* = 0.00026). (Right) Averaged and normalized Max AMPAR-oEPSC/FV ratio in P6 vs. P8 slices (P6: n = 21, P8: n = 19, *p* = 0.0005).

Before commencing with presynaptic genetic manipulations, we first assessed whether our method accurately reflects changes in glutamatergic neurotransmission. To this end, super-resolution images of dendritic spines were obtained via Structured Illumination Microscopy, which revealed that there is a 2.5-fold increase in CA1 pyramidal neuron dendritic spine density in hippocampal slice cultures prepared from postnatal day 8 (P8) pups vs. postnatal day 6 (P6) pups (Fig. 2A). This change in spine number reflects the high level of synaptogenesis occurring in the brain during this time in postnatal development.

The vast majority of glutamatergic synapses are located on dendritic spines^9,10^. Given that glutamatergic synapses can be silent (i.e. lacking AMPARs but expressing NMDARs)^11^ and that presynaptic release probability does not change between P6 and P8 slices based on paired-pulse ratios (Fig. 2B), we reasoned that by using our new method we would resolve a fold change in NMDAR-mediated currents in P8 vs. P6 slices that was similar to the change we observed in dendritic spine density. We first examined the oEPSC’s and the fiber volley amplitudes over a range of light intensities to ensure that, with increasing light intensity, the fiber volley amplitudes and oEPSCs did not change independently of one another. The collected range data consisted of at least 3 light intensity levels per cell. We plotted the oEPSC’s as a function of fiber volley amplitudes and fit the measurements from each cell with a linear regression line. Each linear fit represents a recording from a single neuron. In every recording, we observed a linear relationship between oEPSC and FV amplitudes (Fig. 2C,F). We then averaged the NMDAR-oEPSC/FV ratios in each condition and, remarkably, resolved a 2.5-fold increase in NMDAR-oEPSC/FV amplitude ratio in the P8 slices compared to the P6 slices (Fig. 2E), a near perfect match to the increase in dendritic spine number we observed (Fig. 2A). Furthermore, we assessed whether a single measurement of the NMDAR-oESPC/FV ratio at maximum optical stimulation (Max NMDAR-oEPSC/FV ratio) was sufficient to maintain the accuracy. For each of the recordings plotted in Fig. 2C, a single measurement of NMDAR-oEPSC and FV amplitude was taken for each neuron at maximum optical stimulation and plotted as individual circles in Fig. 2D. We also took Max NMDAR-oEPSC/FV ratios from additional neurons where fewer than 3 light intensity levels were assessed. These Max NMDAR-oEPSC/FV ratios were averaged for each condition, which resulted in a 2.4-fold increase in P8 slices, comparable to the previous measurements (Fig. 2D,E). Such data demonstrate that our method only requires a single oEPSC/FV amplitude ratio per neuron to maintain a high level of accuracy. Using the same approach, we also compared AMPAR-oEPSC/FV ratios in P6 and P8 slices and found a 2.1-fold increase in P8 slices compared to the P6 slices (Fig. 2F,H). Averaging only the Max AMPAR-oEPSC/FV ratios from each cell instead of taking multiple measurements resulted in a 2.2-fold change (Fig. 2G,H), again demonstrating that a single ratio measurement is sufficient for maintaining our method’s accuracy. The smaller fold increase we observe in AMPAR-oEPSC/FV ratio with respect to that of NMDARs is likely explained by the presence of silent synapses. Taken together, the functional NMDAR-oEPSC/FV ratio data from our method were nearly identical to that of dendritic spine analysis and therefore demonstrate that our technique represents a highly accurate method of measuring the strength of synaptic contact between neurons.

### Method Validation 2: Synaptotagmin 1 knockdown

**Figure 3:**
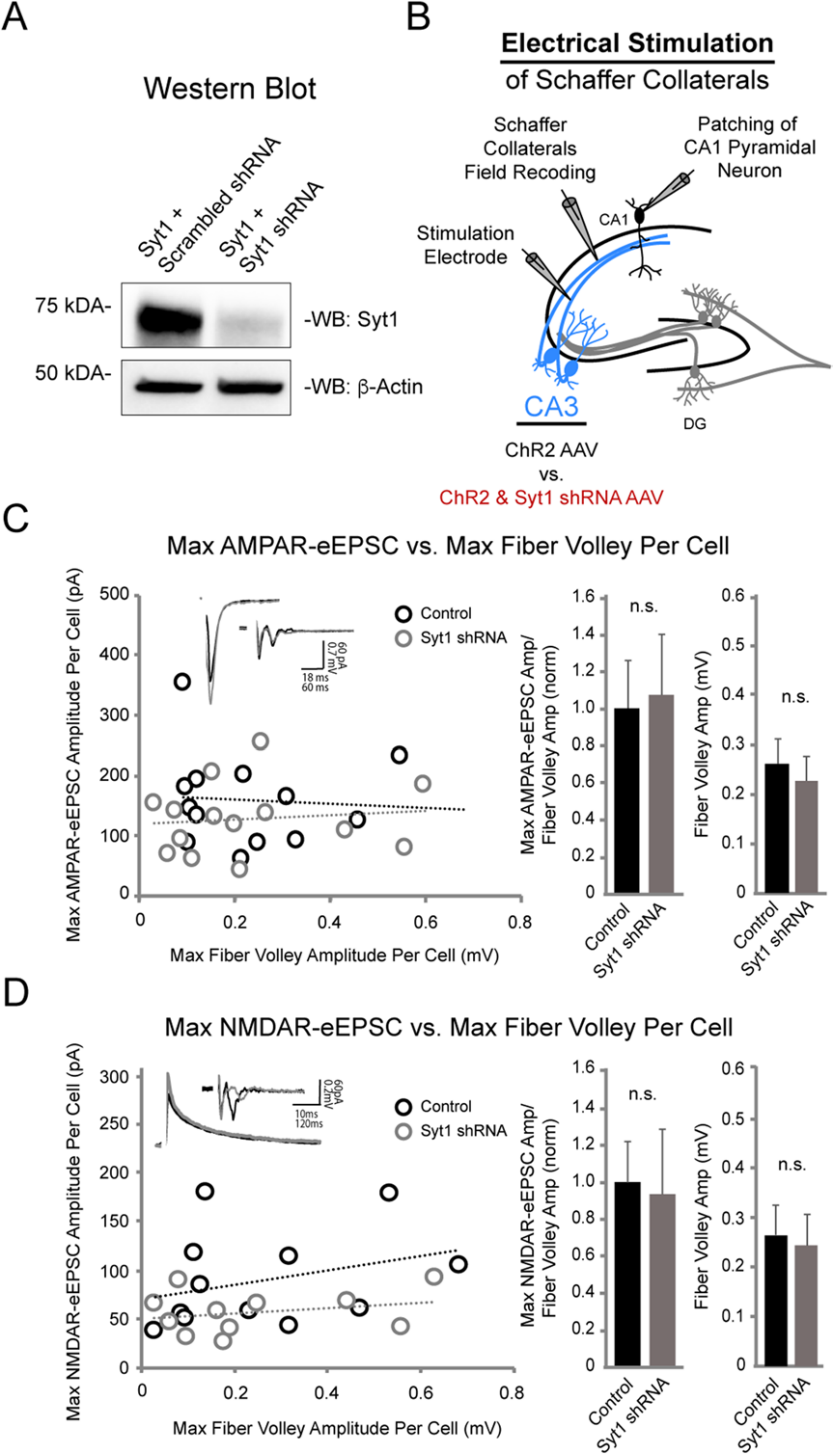
Deficits in CA3-CA1 excitatory synaptic transmission following Syt1 knockdown in CA3 pyramidal neurons are not observed with electrical stimulation. (**A**) Western blot showing knockdown of Syt1 with Syt1 shRNA in HEK293 cells. Top: probed for Syt1, bottom: probed for ß-Actin. The same blot was used to probe for both proteins. Images have been cropped for clarity. Full-length blots are presented in Supplementary Fig. 1. (**B**) Schematic illustration of recording setup using electrical stimulation. AAV expressing either ChR2 only (control) or ChR2 with Syt1 shRNA were injected into CA3 pyramidal neurons, and Schaffer collaterals were electrically stimulated with an electrode. Schaffer collateral field recordings and whole-cell patch recordings of CA1 pyramidal neurons were simultaneously acquired. In (**C**,**D**), Wilcoxon rank sum test was used. * p < 0.05, n.s., not significant. (**C**) (Left) Max AMPAR-eEPSC/FV ratios per cell in control vs. Syt1 shRNA conditions following electrical stimulation. Each point represents the Max AMPAR-eEPSC/FV amplitude ratio of one cell; measurements from each condition were fitted with a linear regression line. Insets show representative traces from control (black) and Syt1 shRNA (gray) conditions with stimulation artifacts removed. (Center) Averaged and normalized Max AMPAR-eEPSC/FV amplitude ratio in control vs. Syt1 shRNA conditions following electrical stimulation (control n = 14, Syt1 shRNA n = 14, *p* = 0.31). (Right) Averaged fiber volley amplitudes in control vs. Syt1 shRNA conditions (control n = 14, Syt1 shRNA n = 14, *p* = 0.51). (**D**) (Left) Max NMDAR-eEPSC/FV ratios per cell in control vs. Syt1 shRNA conditions following electrical stimulation. Each point represents the Max NMDAR-eEPSC/FV amplitude ratio of one cell; measurements from each condition were fitted with a linear regression line. Insets show representative traces from control (black) and Syt1 shRNA (gray) conditions with stimulation artifacts removed. (Center) Averaged and normalized Max NMDAR-eEPSC/FV amplitude ratio in control vs. Syt1 shRNA conditions following electrical stimulation (control n = 12, Syt1 shRNA n = 11, *p* = 0.12). (Right) Averaged fiber volley amplitudes in control vs. Syt1 shRNA conditions (control n = 12, Syt1 shRNA n = 11, *p* = 0.79).

Next, to establish that this method allows for a new and accurate approach to studying presynaptic genetic manipulations, we examined the effects of knocking down (KD) synaptotagmin 1 (Syt1) in the CA3 pyramidal neurons. Syt1 is a well-established calcium sensor on presynaptic vesicles, involved in synchronous vesicular neurotransmitter release and rapid synaptic transmission^12^. The role of presynaptic Syt1 in glutamatergic neurotransmission has been examined in dissociated cortical cultures^13,14^, hippocampal cultures^15^, dentate granule cell-basket cell synapses^16^, and CA1-Subiculum synapses^15,17^. Surprisingly, the role of Syt1 at the CA3-CA1 synapse, one of the most studied synapses in the brain, has not yet been explored. Using an AAV co-expressing ChR2 and Syt1 shRNA, we knocked down Syt1 expression in CA3 pyramidal neurons. An AAV expressing only ChR2 (control), as well as an AAV co-expressing ChR2 and a scrambled shRNA, were each injected into the CA3 region of the two control conditions. The Syt1 shRNA was previously validated^17^ and we verified via western blot that it significantly reduced Syt1 levels in HEK293 cells compared to a scrambled shRNA (Fig. 3A and S1). To test if the optogenetic component was necessary, as well as whether electrical stimulation of Schaffer collaterals would be sufficient to detect an alteration in synaptic efficacy with Syt1 KD, we first electrically stimulated Schaffer collaterals (Fig. 3B) and found no change in either Max AMPAR- or Max NMDAR-electrically evoked excitatory post synaptic current/fiber volley (AMPAR- or NMDAR-eEPSC/FV) amplitude ratios between Syt1 KD and control conditions (Fig. 3C,D). We speculated that the indiscriminate electrical stimulation of Schaffer collaterals from both virally-transduced Syt1 KD and untransduced CA3 pyramidal neurons may have masked a change in synaptic transmission.

**Figure 4:**
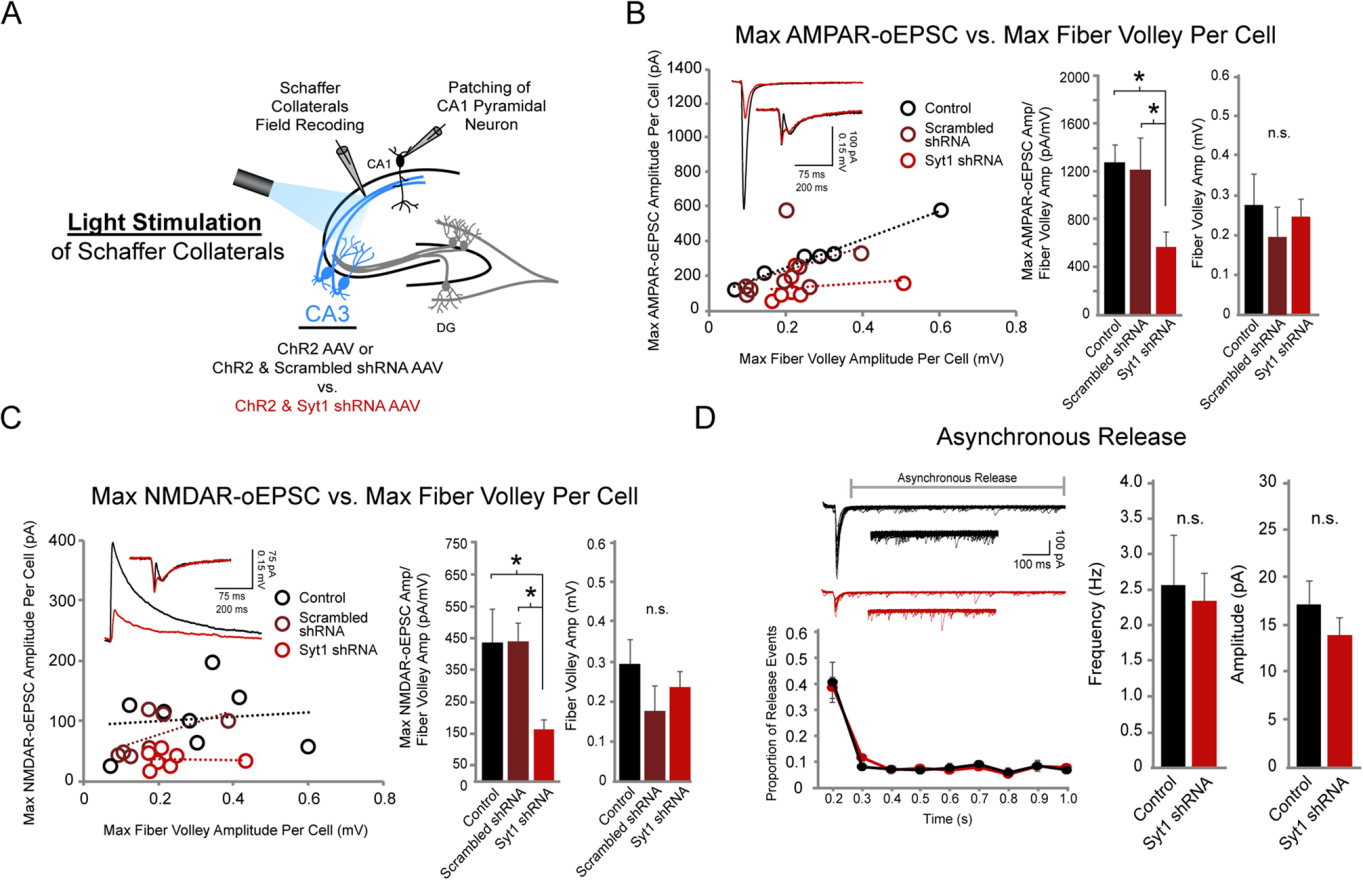
Syt1 knockdown diminishes CA3-CA1 excitatory synaptic transmission with optical stimulation of CA3 pyramidal neurons. (**A**) Schematic illustration of recording setup using optogenetic stimulation. AAV expressing: (1) ChR2 only (control), (2) ChR2 with scrambled shRNA (scrambled shRNA), or (3) ChR2 with Syt1 shRNA were injected into the CA3 region of hippocampal slices. Schaffer collaterals were optically stimulated with blue light. Schaffer collateral field recordings and whole-cell patch recordings of CA1 pyramidal neurons were simultaneously acquired. For (**B**)–(**D**), Wilcoxon rank sum test was used. * p < 0.05, n.s., not significant. (**B**) (Left) Max AMPAR-oEPSC/FV ratios per cell in control, scrambled shRNA & Syt1 shRNA conditions following optical stimulation. Each point represents the maximum AMPAR-oEPSC/FV amplitude ratio of one cell; measurements from each condition were fitted with a linear regression line. Insets show representative traces from control (black) and Syt1 shRNA (red) conditions. (Center) Averaged and normalized Max AMPAR-oEPSC/FV amplitude ratio in control, scrambled shRNA & Syt1 shRNA conditions (n = 6, 8, 7 respectively, control vs. Syt1 shRNA *p* = 0.007, scrambled vs. Syt1 shRNA *p* = 0.0037). (Right) Averaged fiber volley amplitudes in control, scrambled shRNA & Syt1 shRNA conditions (control n = 6, scrambled shRNA n = 8, Syt1 shRNA n = 7, control vs. Syt1 shRNA *p* = 0.63, scrambled vs. Syt1 shRNA *p* = 0.46). (**C**) (Left) Maximum NMDAR-oEPSC/FV ratios per cell in control, scrambled shRNA & Syt1 shRNA conditions. Insets show representative traces from control (black) and Syt1 shRNA (red) conditions. (Center) Averaged and normalized NMDAR-oEPSC/FV amplitude ratio in control, scrambled shRNA & Syt1 shRNA conditions (n = 8, 7, 7 respectively, control vs. Syt1 shRNA *p* = 0.022, scrambled vs. Syt1 shRNA *p* = 0.0023). (Right) Averaged fiber volley amplitudes in control, scrambled shRNA & Syt1 shRNA conditions (n = 8, 7, 7 respectively, control vs. Syt1 shRNA *p* = 0.46, scrambled vs. Syt1 shRNA *p* = 0.12). (**D**) Asynchronous release following optically-induced synchronous release in control (black) vs. Syt1 shRNA (red) conditions. (Left, Top) Representative traces, 10 sweeps of release events merged to show individual release events. (Left, Bottom) Proportion of release events over one second following initial optical stimulus. (Right) Frequency and amplitudes of asynchronous release events in control vs. Syt1 shRNA conditions (n = 6 each condition, both frequency and amplitude, *p* = 0.9372 and 0.4848 respectively).

Using the same experimental setup, we then optically stimulated the Schaffer collaterals in order to selectively stimulate CA3 pyramidal neurons co-expressing ChR2 and Syt1 shRNA (Fig. 4A). In marked contrast to electrical stimulation, optical stimulation revealed a 60% reduction in the Max AMPAR-oEPSC/FV ratio and a 55% reduction in the Max NMDAR-oEPSC/FV ratio in the Syt1 KD condition compared to both the control and the scrambled shRNA conditions (Fig. 4B,C). Comparable ranges of optical axonal stimulation were used in all conditions as shown by similar average fiber volley amplitudes (Fig. 4B,C). The similar reductions we observe in Max AMPAR- and NMDAR-oEPSC/FV ratios are consistent with reduced Syt1 expression in CA3 pyramidal neurons inhibiting evoked glutamate release onto both glutamate receptor subtypes. We also examined whether asynchronous release was affected by knocking down Syt1 in CA3 pyramidal neurons. It is generally believed that Syt1 plays a selective role in fast, synchronous release, and that removing Syt1 does not interfere with normal asynchronous release^13,17^. As expected, we found no significant differences in the frequency or amplitude of asynchronous release events with Syt1 knockdown compared to the control condition (Fig. 4D). Altogether, our results demonstrate that Syt1 plays a significant role in synchronous glutamate release at CA3-CA1 synapses. Furthermore, our side-by-side experiments comparing electrical vs. optical stimulation in the same setup demonstrate the utility of our new method, given that electrical stimulation failed to resolve a change in glutamate release following CA3 pyramidal neuron transduction with the Syt1 RNAi.

## Discussion

Historically, the ease of studying the contribution of presynaptic proteins to synaptic regulation has lagged behind the study of synaptic regulation by postsynaptic proteins. Isolation of genetic manipulations to specific populations of presynaptic neurons often comes with great cost, and precise methods of quantifying the effects these manipulations have on synaptic efficacy are sorely lacking. Isolating the presynaptic function of a protein in glutamatergic synapses has thus far been predominantly limited to two common approaches: dissociated neurons in culture and transgenic mouse lines. Using dissociated neurons is a straightforward system for making genetic manipulations, but its critical limitation stems from the fact that cultured neurons do not maintain their original, endogenous synaptic circuitry. Instead, cultured neurons form indiscriminate synaptic connections regardless of their endogenous connections or cell type. The hippocampus, for example, has discrete regions of neuronal subtypes such as CA3 and CA1 pyramidal neurons and dentate granule neurons, and it has been increasingly appreciated that there is pathway-specific regulation that functionally differentiates these synapses^5,18–21^. Our novel approach allows for keeping the endogenous circuitry of synapses intact by utilizing hippocampal slices. In cultured hippocampal slice preparations, genetic manipulations can easily be targeted to the CA3 subregion, and whole-cell recordings from CA1 pyramidal neurons allow the experimenter to assess the consequences of the presynaptic manipulation at CA3-CA1 synapses with a high level of confidence.

Currently the most sophisticated approach for studying presynaptic regulation requires generating knockout mouse lines. Generation of such mouse lines is not a trivial process and can come at great cost in terms of both money and time. However, one potential problem with using this approach is that global knockout models limit the ability to discern whether any observed phenotype is due to a pre- or postsynaptic role of the protein. In order to conclusively identify the presynaptic function of a protein in transgenic mice, the genetic manipulation must be limited to a specific population of presynaptic neurons. For example, to study the presynaptic function of synaptotagmin 1 using transgenic mice in CA3-CA1 synapses, the genetic manipulation must be limited to CA3 pyramidal neurons. This isolated manipulation is especially important given that canonical presynaptic proteins such as synaptotagmins have also been shown to play postsynaptic roles in synaptic regulation^22^. Furthermore, germline knockout models can be lethal. Even if the mouse is viable, the protein is absent throughout development, and this can lead to compensatory mechanisms and downstream effects that hinder our ability to isolate the specific role the protein plays at the synapse. Our approach not only allows for a more efficient way of selectively manipulating presynaptic neurons genetically, but also allows the observation of the immediate consequences of the genetic manipulation which reduces the likelihood of compensatory mechanisms occluding changes in phenotype.

In the present study we validated our novel method by accurately matching results from dendritic spine analysis comparing cultured hippocampal slices from P6 and P8 rat pups. During this period of rapid synaptogenesis, there was a significant increase in spine density, which our metric mirrored remarkably well. We then utilized the method to study the presynaptic role of Syt1 in the CA3-CA1 synapse for the first time. Based on previous literature, we speculated that knocking down expression of Syt1 in CA3 pyramidal neurons would disrupt transmission in the CA3-CA1 synapses, and indeed we found a significant reduction in the oEPSC/FV ratios for both AMPAR- and NMDAR-mediated currents. A complete absence of synchronous release, however, has been reported in Syt1 knockout models at other synapses. The remaining synchronous release we observe at CA3-CA1 synapses following Syt1 knockdown may be supported by either residual Syt1 protein or additional synaptotagmin-related proteins. While we used an RNAi to influence protein expression in the present study, it is important to note that our method can be used with other forms of genetic modification that lead to complete elimination of protein expression (e.g. CRISPR-based strategies, CRE/lox mouse lines, etc.). In our study we found a similar level of reduction in both AMPAR- and NMDAR-mediated currents following presynaptic knockdown of Syt1, which would be expected from a synapse where glutamate release is inhibited. Consistent with previous reports^13,17^, we also find no change in asynchronous release properties with reduced Syt1 expression. In future experiments using our new method, it will be interesting to explore the roles of other synaptotagmin isoforms at CA3-CA1 synapses.

A time- and cost-effective approach to rigorously study the molecular regulation of presynaptic function within a native mammalian synapse had yet to exist prior to this study. Our hope is that this novel approach will lead to rapid advancements in understanding molecular regulation of presynaptic function. Furthermore, the utility of our method can be expanded by incorporating biolistic transfection of postsynaptic neurons for simultaneous pre- and postsynaptic genetic control (Fig. 1A). By genetically controlling each side of the synapse independently, this combination of methods will open doors for a new line of synaptic studies such as investigating the roles of specific isoform combinations of transsynaptic adhesion molecules. Transsynaptic adhesion molecules regulate synapse development, as well as play a role in synaptic transmission and plasticity^23^. In the hippocampus, postsynaptic dendritic spines with larger PSDs and higher surface AMPAR expression have corresponding presynaptic terminals with larger active zones and more docked vesicles. Such findings demonstrate the importance of pre- and postsynaptic coordination, most likely mediated by trans-synaptic adhesion molecules^23–25^. These proteins, which include cadherins, ephrins, neuroligins, and neurexins, are especially important to investigate given that many have been strongly implicated in cognitive disorders such as autism and schizophrenia^26–29^. However, these proteins are difficult to efficiently study because they have multiple isoforms, with neurexin alone having thousands of potential splice variants^30,31^. Our new method can circumvent existing methods’ limitations, expedite the exploration of these proteins, and ultimately lead to enhancing our understanding of the synapse.

## Methods

### Experimental constructs

pAAV.CAG.hChR2(H134R)-mCherry.WPRE.SV40 and the AAV9 viral particles produced from the plasmid were a gift from Karl Deisseroth (Addgene viral prep #100,054-AAV9). Syt1 shRNA target sequence 5’-GAGCAAATCCAGAAAGTGCAA-3’ was previously determined and validated^17^. pAAV.H1.Syt1shRNA.CAG.hChR2(H134R)-mCherry was constructed and packaged by VectorBuilder by modifying the original hChR2 pAAV plasmid to include the Syt1 shRNA behind an H1 promoter (Vector ID VB170324-1065bbv).

pAAV.H1.scrambledshRNA.CAG.hChR2(H134R)-mCherry was also constructed and packaged by VectorBuilder by modifying the Syt1 shRNA AAV plasmid to instead express a scrambled shRNA (Vector ID VB191113-1716anp). Rat synaptotagmin 1 in pcDNA3.1^+^/C-(K)DYK was acquired from GenScript (NM_001033680.2). pFUGW-GFP was used to identify transfected neurons in spine density analysis.

### Slice virus injection and electrophysiology

All experimental procedures were carried out in accordance with the National Institutes of Health (NIH) Guide for the Care and Use of Laboratory Animals and approved by the University of Southern California Institutional Animal Care and Use Committee. This study was carried out in compliance with the ARRIVE guidelines. 400 μm rat organotypic hippocampal slice cultures were prepared from both male and female P6 to P8 Sprague Dawley rats as previously described^32–34^. AAV9 viral particles from pAAV plasmids expressing channelrhodopsin (ChR2(H134R)-mCherry only, Syt1 shRNA + ChR2(H134R)-mCherry, or scramble shRNA + ChR2(H134R)-mCherry) were injected into the CA3 pyramidal layer of the organotypic hippocampal slice cultures on DIV1 using a Nanoject II device (Drummond Scientific). Successful viral transduction was later verified by mCherry epifluorescence in the CA3 region and particularly in the Schaffer collaterals (Fig. 1A). Culture media was exchanged every other day until recording on DIV12-13. During recordings, slices were maintained in room-temperature artificial cerebrospinal fluid (aCSF) external solution containing (in mM): 119 NaCl, 2.5 KCl, 1 NaH_2_PO_4_, 26.2 NaHCO_3,_ 11 glucose, 4 CaCl_2_, and 4 MgSO_4_. 5 μM 2-chloroadenosine and 0.1 mM picrotoxin were also added to the aCSF to dampen epileptiform activity and block GABA_A_ receptor activity, respectively. Osmolarity was adjusted to 310–315 mOsm. aCSF was saturated with 95% O_2_/5% CO_2_ throughout recording. Borosilicate extracellular field recording electrodes were filled with the same aCSF external solution. Borosilicate whole-cell recording electrodes were filled with an internal, whole-cell recording solution containing (in mM): 135 CsMeSO_4_, 8 NaCl, 10 HEPES, 0.3 EGTA, 5 QX-314, 4 Mg-ATP, and 0.3 Na-GTP. The internal solution was adjusted to pH 7.3–7.4 and osmolarity of 290–295 mOsm.

Virally transduced Schaffer collaterals were either electrically stimulated with a monopolar glass electrode filled with aCSF or optically stimulated with blue light to evoke a postsynaptic response in CA1 pyramidal neurons. Whole-cell recordings of a CA1 pyramidal neuron’s AMPAR- and NMDAR-mediated current amplitudes were made simultaneously with extracellular field recordings in the Schaffer collaterals (Fig. 1C). Synaptic responses were acquired using a Multiclamp 700B amplifier (Molecular Devices). AMPAR-mediated EPSCs were measured at -70 mV. NMDAR-mediated EPSCs were measured at + 40 mV, temporally isolated from AMPAR currents by measuring amplitudes 250 ms after stimulus onset. In most cases, AMPAR- and NMDAR-mediated currents were recorded from the same neuron by changing the membrane potential. Optically-induced extracellular field recording trace was merged with the postsynaptic whole-cell recording trace to verify that the postsynaptic responses (field EPSP and oEPSC) were aligned in time and also to isolate the presynaptic fiber volley (Fig. 1D). It was demonstrated that with increased optical stimulation strength, there was a corresponding increase in both presynaptic fiber volley amplitude and postsynaptic current amplitude (Fig. 1E). No more than one simultaneous recording was performed on any given hippocampal slice. To assess asynchronous release, asynchronous EPSCs with an amplitude of ≥ 5 pA and a rate of rise of ≥ 4pA/ms were automatically detected and analyzed with customized IGOR software^35^.

### Immunoblotting

To validate the Syt1 shRNA construct, HEK293 cells were co-transfected with a rat Syt1 construct and pAAV.H1.Syt1shRNA.CAG.hChR2(H134R)-mCherry using Lipofectamine 2000. Syt1 co-transfected with pAAV.H1.ScrambleshRNA.CAG.hChR2(H134R)-mCherry was used as the control. Lysates were prepared 72 h post transfection and lysed in RIPA buffer containing protease inhibitor mix (Thermo Scientific, Halt Protease Inhibitor Cocktail). Proteins were resolved by SDS-PAGE and analyzed by western blot with antibodies against rat synaptotagmin 1 (1:1000, Synaptic Systems Cat#105 011) and β-actin (1:1000, Cell Signaling Technology Cat#4970). Secondary antibodies used were anti-mouse and anti-rabbit IgG, HRP-linked antibodies, respectively (1:10,000, Cell Signaling Technology Cat#7076/7074).

### Spine density analysis

CA1 pyramidal neurons in cultured hippocampal slices prepared from P6 and P8 rat pups were biolistically transfected with a pFUGW-GFP construct on DIV1. On DIV7, slices were fixed in 4%PFA/4% sucrose in PBS, washed 3 times in PBS, and cleared using an abbreviated SeeDB-based protocol^36^. Images were acquired using super-resolution microscopy (Elyra Microscope System, Zeiss), blinded to condition, with an oil-immersion 100 × objective lens. Image acquisition and analysis were carried out as described previously^2,4^.

### Statistical analysis

Electrophysiological recordings of normalized EPSC amplitudes/FV amplitudes, paired pulse facilitation, and asynchronous release were analyzed using a Wilcoxon Rank Sum Test. Data analysis was performed using Igor Pro (Wavemetrics). Spine density data was analyzed using unpaired Student’s *t*-test. P-value of < 0.05 was considered statistically significant. Error bars represent standard error of the mean measurement. Sample sizes in the present study are similar to those reported in the literature^37,38^.

## Supplementary Information

Supplementary Information.

## Data Availability

All data generated and analyzed during this study are available from the corresponding author upon reasonable request.
