# Supplementary material for: An optogenetic method for investigating presynaptic molecular regulation
Source: Sci Rep. 2021 May 31;11:11329. doi: 10.1038/s41598-021-90244-0 (PMC8166971; doi:10.1038/s41598-021-90244-0)
Supplement: Supplementary file 1 — Supplementary Information. [file 41598_2021_90244_MOESM1_ESM.pdf]

An optogenetic method for investigating presynaptic molecular regulation

Yuni Kay[1] and Bruce E. Herring[1,2*]

[1]Neuroscience Graduate Program, University of Southern California, Los Angeles, CA 90089, USA.
[2]Department of Biological Sciences, Neurobiology Section, Dornsife College of Letters, Arts and Sciences, University of Southern California, Los Angeles, CA 90089, USA.

*Correspondence: bherring@usc.edu (B.E.H)

Full Western Blots

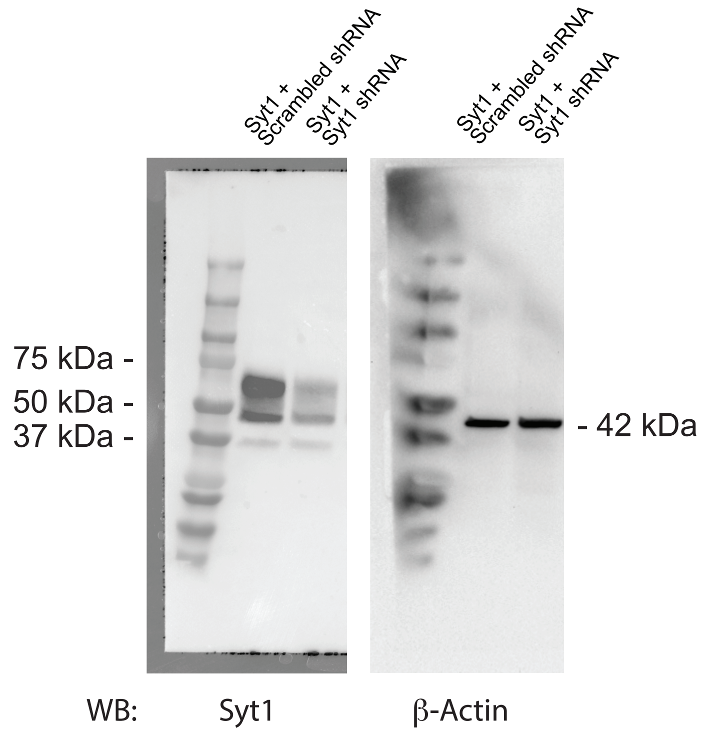

Supplementary Figure 1: Full western blots showing knockdown of Syt1 with Syt1 shRNA. The same blot is shown in both images, probed first for Syt1 (left), stripped, and probed for β-Actin (right). First lane: Syt1 and scrambled shRNA co-expressed in HEK293 cells. Second lane: Syt1 and Syt1 shRNA co-expressed in HEK293 cells. In the Syt1 western blot, the 60kD band likely represents the mature, fully glycosylated form of Syt1 and the 47kDa band the immature form of Syt1 (Atiya-Nasagi et al., *Journal of Cell Science*, 2005).
